# Alternative measures of body composition and wage premium: New evidence from Indonesia

**DOI:** 10.1371/journal.pone.0219438

**Published:** 2019-08-09

**Authors:** Md Nazmul Ahsan, Petri Böckerman

**Affiliations:** 1 Department of Economics, Saint Louis University, Saint Louis, Missouri, United States of America; 2 University of Jyväskylä, Jyväskylä, Finland; 3 Labour Institute for Economic Research, Helsinki, Finland; 4 IZA Institute of Labor Economics, Bonn, Germany; TED University, TURKEY

## Abstract

This paper examines the relationship between body composition and earnings in a developing country setting. We use body mass index, waist circumference and hip circumference. Exploiting the panel structure of our longitudinal survey, we find that along with BMI, waist circumference is related to higher earnings in Indonesia.

## Introduction

The link between health and earnings is a widely researched topic. While in the developed countries a higher Body Mass Index (BMI) is negatively related to earnings [1–2], in the developing countries the same relationship is found to be positive [3]. However, research in medical studies indicates that BMI, alone, is not a sufficient measure of body composition [4]. The alternative measures may also be relevant; the earlier empirical literature using the measures of body composition in the developed countries has found that the effect of obesity on labor market outcomes is not identical using different measures of obesity [5–8]. The links between income and body weight are potentially complicated. Lakdawalla and Philipson [9] argue that income may have an inverted U-shaped relationship with body weight.

This paper examines the role of BMI as well as other relevant measures of body composition, waist circumference and hip circumference, on labor earnings in Indonesia. Our analyses are closely related to the earlier empirical literature. First, the measures of body composition have been used in the earlier literature on wage penalty of obesity using data also from other developing countries (e.g. [10–15]). Second, we report results from quantile regression models that have also been used in the related earlier literature (e.g. [13], [15]).

We use a rich longitudinal data—the Indonesian Family Life Survey. Because of the panel structure, we are able to empirically estimate the relationship between wage and different measures of body composition (BMI, waist circumference, hip circumference) with or without individual fixed effects. Our strongest empirical specification is the model that accounts for individual fixed effects. We find a positive relationship between BMI and labor income. When we include waist circumference in the regression model, we observe an attenuation of the association between BMI and labor income. In the model without individual fixed effects, the relationship between BMI and labor income does not statistically differ from zero. In the model with fixed effects, the BMI remains a significant predictor of earnings. The quantile regression results do not reveal notable differences in relationships for low earners vs. high earners.

## Data and empirical specifications

The Indonesian Family Life Survey (IFLS) is an ongoing longitudinal survey. The IFLS has randomly chosen over 300 communities from 13 provinces. After the first wave in 1993, the second, third, fourth and fifth waves have taken place in 1997, 2000, 2007 and 2014 respectively. In the first wave about 22,000 individuals from 7,224 households were interviewed. An appealing feature of the IFLS is its low attrition rate [16].

Another advantage of the IFLS data set is that it collects different types of health measures. The availability of different measures, however, vary across waves and age groups. For example, height and weight measures for all individuals are available in all waves, but the waist and hip circumference data are available only for the last three waves for individuals who are 40 years and older. Height, weight, waist and hip circumferences were measured by a health professional (typically a nurse) [16].

Since we interested in analyzing the relationship between wage and various indicators of body composition (BMI, waist circumference, hip circumference), we restrict our sample to third to fifth wave. Moreover, we further restrict our samples to men who are earning a monthly wage or salary and aged between 40 to 55. The age of retirement in Indonesia is 56. Moreover, height shrinkage occurs at an older age due to aging; including older individuals in the sample may lead to an incorrect measurement of body mass index (BMI). We exclude women because labor force participation among female is low Indonesia. According to Schaner and Das [17], only 32.3% women were employed as wage worker in Indonesia in 2012. To compare the wages across the waves, we convert the wages in real wages using the 1993 base year. We then limit our sample to men who have health and wage information at least in two survey waves out of the last three last survey waves. We are using individual fixed effects in variants of our empirical model. Restricting the sample to individuals, who have data for at least two times, allows us to estimate changes within the individuals over time.

The data also have another minor limitation; a few of the health measures take extremely low values. For example, the height of one individual was reported as low as 52 centimeters. Moreover, some weight measures of adults were reported as low as 5 kg. Following Huang et al. [18], we drop men with height below 120 centimeters. As a result, we only lose one individual from the sample. Furthermore, for weight, waist circumference, hip circumference we winsor the values at 0.01 percent level. This ensures that our point estimates are not driven by extremely low values.

Table 1 presents the summary statistics. On average, the sampled individuals are about 47 years old and have about 8.6 years of education. The average body mass index (BMI) is 23.47, average height is 161.93 centimeters, the average waist circumference is 83.05 centimeters, and the average hip circumference is 91.57 centimeters. Table 1 also shows that both paternal and maternal education of these individuals are very low.

**Table 1:** Summary statistics.

Variable	Observations	Mean
Log Real Net Wage/ Salary: Last Month	1921	5.44
Age at Survey	1921	47.14
Years of Education	1921	8.60
Body Mass Index	1921	23.47
Height (cm)	1921	161.93
Waist circumference (cm)	1921	83.05
Hip Cirumference (cm)	1921	91.57
Father’s Years of Education	1921	3.00
Mother’s Years of Education	1921	1.93

Source: The Indonesian Family Life Survey.

We estimate specifications of the form:

$$Y_{it}=\beta +W_{it}\gamma +Z_{i}\theta +X_{it}\eta +\delta_{t}+\varepsilon_{it}$$ (1)

We study the earnings outcomes Y for an individual i. W is the vector of body composition; it includes BMI, waist and hip circumference. The vector Z represent time invariant individual characteristic—years of education. The vector X represent time variant individual characteristics—age at the time of survey. δ_t_ represent wave fixed effects where t is the wave number. In another variation of regression, we also include α_i_—individual fixed effects—in the regression equation above. Naturally, when we include individual effects, we are unable to estimate θ.

## Results

We first estimate a set of models without using individual-level fixed effects (Table 2). The estimates in Column 2 replicate the earlier findings between BMI and wage. Following the advice in Abadie et al. [19], we report standard errors that are clustered at the community ID level, because community ID level is the level of sampling design. We find that BMI is significantly related to higher level of earnings in Indonesia. The quantitative size of the estimate is substantial. The point estimate shows one-unit increase in BMI is associated with 3.3% higher earnings.

**Table 2:** Estimation results without individual fixed effects.

	(1)	(2)	(3)	(4)
Age at Survey	0.015***	0.014***	0.012**	0.012**
	(0.005)	(0.005)	(0.005)	(0.005)
Years of Education	0.101***	0.093***	0.091***	0.091***
	(0.004)	(0.004)	(0.004)	(0.004)
Body Mass Index		0.033***	0.008	0.007
		(0.005)	(0.007)	(0.007)
Waist circumference (cm)			0.011***	0.011***
			(0.003)	(0.003)
Hip circumference (cm)				0.001
				(0.003)
R^2^	0.347	0.369	0.376	0.376
Observations	1894	1894	1894	1894

Notes: The dependent variable is earnings. Earnings are measured as the log of last month wage. Standard errors clustered error at the community ID level: * statistically significant at the 0.10 level

** at the 0.05 level

*** at the 0.01 level.

Next, we proceed to add waist circumference and hip circumference to the model in Columns 3–4. These results show that waist circumference is positively related to earnings. The size of the coefficient implies that one additional centimeter of waist circumference is associated with roughly a 1% increase in earnings. BMI is no longer a statistically significant determinant of earnings.

A critical issue in the results presented in Table 2 is that there are arguably individual-level unobservable characteristics such as personality traits, which may influence both the measures of body composition and wage. Moreover, the unobserved time-invariant health conditions may also affect selection into the labor force.

To mitigate these concerns, we exploit the panel structure of our data by including individual-level fixed effects to the model (Table 3). We observe that both BMI and waist circumference are predictors of labor income. However, similar to our model without individual fixed effects, we find that the inclusion of waist circumference in the model weakens the association between BMI and labor income. The point estimate of waist circumference is slightly lower than in the model that does not absorb individual-level fixed effects. BMI and waist circumference remain statistically significant at the 10% level. The exact p-values for BMI and waist circumference are 0.062 and 0.098, respectively.

**Table 3:** Estimation results with individual fixed effects.

	(1)	(2)	(3)	(4)
Age at Survey	-0.010	-0.007	-0.008	-0.008
	(0.033)	(0.033)	(0.033)	(0.032)
Years of Education	.	.	.	.
Body Mass Index		0.030***	0.019*	0.020*
		(0.010)	(0.011)	(0.010)
Waist circumference (cm)			0.007*	0.008*
			(0.004)	(0.005)
Hip circumference (cm)				-0.001
				(0.005)
R^2^	0.831	0.833	0.834	0.834
Observations	1894	1894	1894	1894

Notes: The dependent variable is earnings. Earnings are measured as the log of last month wage. Standard errors clustered at the community ID level.

* statistically significant at the 0.10 level

** at the 0.05 level

*** at the 0.01 level.

We have also estimated models using quantile regressions (Table 4). We use the *xtqreg* command in Stata to estimate fixed effects models using technique by Machado and Santos Silva [20]. Contrary to our expectation, these results show no notable differences in relationships for low earners vs. high earners. The average effect on earnings is not mostly due to effects at the lowest decile.

**Table 4:** Quantile regression results.

	(1)	(2)	(3)
	Quantile (0.25)	Quantile (0.5)	Quantile (0.75)
Age at Survey	-0.009	-0.008	-0.007
	(0.030)	(0.021)	(0.028)
Years of Education	.	.	.
Body Mass Index	0.020**	0.020***	0.019**
	(0.008)	(0.006)	(0.008)
Waist Circumference (cm)	0.008**	0.008***	0.008**
	(0.004)	(0.003)	(0.004)
Hip Circumference (cm)	-0.001	-0.001	-0.001
	(0.004)	(0.003)	(0.004)
R^2^			
Observations	1894	1894	1894

Notes: The dependent variable is earnings. Earnings are measured as the log of last month wage. Standard errors are calculated using bootstrapping (1000 replications).

* statistically significant at the 0.10 level

** at the 0.05 level

*** at the 0.01 level.

## Conclusion

This paper provides new evidence for the existence of wage premium using data from a developing country. Our results show that along with BMI, waist circumference is related to higher earnings in Indonesia. The findings of this paper indicate that the alternative measures of body composition are important predictors of wage premium. A plausible explanation for our findings is that people with lower BMI, suffering from under-nutrition, face wage penalty and overweight people earn wage premium [21]. Reverse causality is also plausible i.e. getting a job increases income, which then makes it easier to buy food and gain weight.

There are two important limitations regarding our results. First, the results are based on male individuals who are between 40 and 55. Therefore, we unable to generalize the results for the overall population. Second, we have only studied the association between measures of body composition and labor income. Although individual fixed effects absorb unobserved time-invariant health conditions as well as preferences, we are unable to control for time-varying unobserved traits.
